# Supplementary material for: Ceramide sensing by human SPT-ORMDL complex for establishing sphingolipid homeostasis
Source: Nat Commun. 2023 Jun 13;14:3475. doi: 10.1038/s41467-023-39274-y (PMC10261145; doi:10.1038/s41467-023-39274-y)
Supplement: Supplementary file 1 — Supplementary Information [file 41467_2023_39274_MOESM1_ESM.pdf]

Supplementary Information

Ceramide sensing by human SPT-ORMDL complex for establishing sphingolipid homeostasis

Tian Xie[1,*], Peng Liu[1,*], Xinyue Wu[1], Feitong Dong[1], Zike Zhang[1], Jian Yue[1], Usha Mahawar[2], Faheem Farooq[2], Hisham Vohra[2], Qi Fang[1], Wenchen Liu[1], Binks W. Wattenberg[2,#] and Xin Gong[1,#]

[1]Department of Chemical Biology, School of Life Sciences, Southern University of Science and Technology, Shenzhen, Guangdong 518055, China.

[2]Department of Biochemistry and Molecular Biology, Virginia Commonwealth University School of Medicine, Richmond, VA 23298, USA.

[*]These authors contributed equally to this work.

[#]Corresponding author. Email: brian.wattenberg@vcuhealth.org (B.W.W.) or gongx@sustech.edu.cn (X.G.)

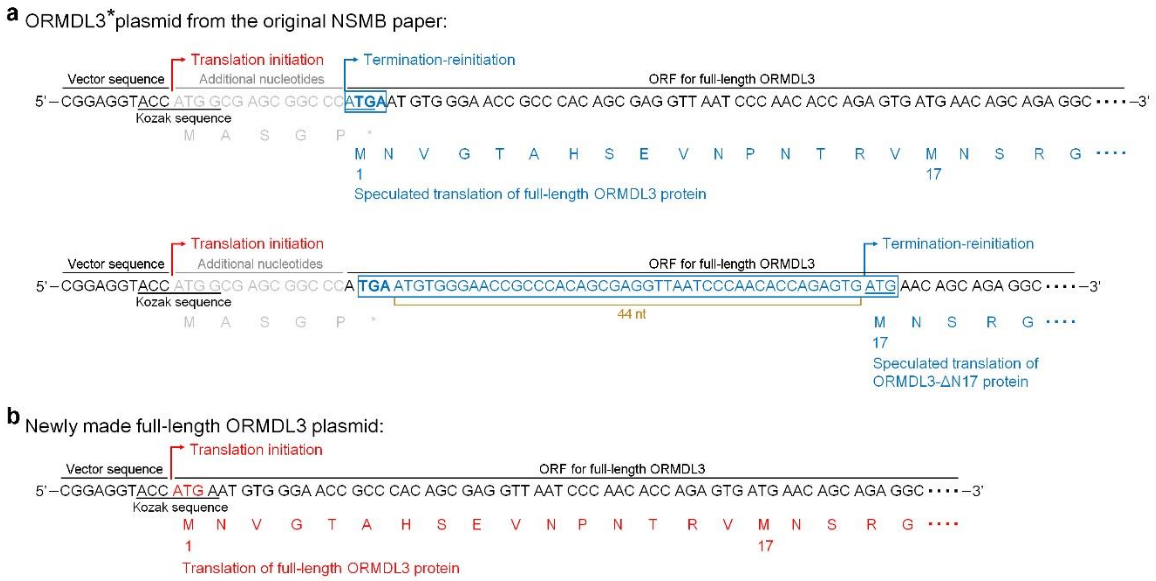

Supplementary Fig. 1 | The sequences of ORMDL3 expression plasmids in previous and current studies. **a,** The ORMDL3* plasmid used in the previous study (ref[27]), which contains 14 extra nucleotides upstream of the ORMDL3 ORF. A possible explanation for the translation of a mixture of the minor full-length ORMDL3 protein and the major N-terminus truncated ORMDL3 protein, which might be caused by a potential premature termination and reinitiation process, is outlined here. **b,** The newly made full-length ORMDL3 plasmid in the current study, which lacks the extra 14 nucleotides upstream of the ORMDL3 ORF in the ORMDL3* plasmid.

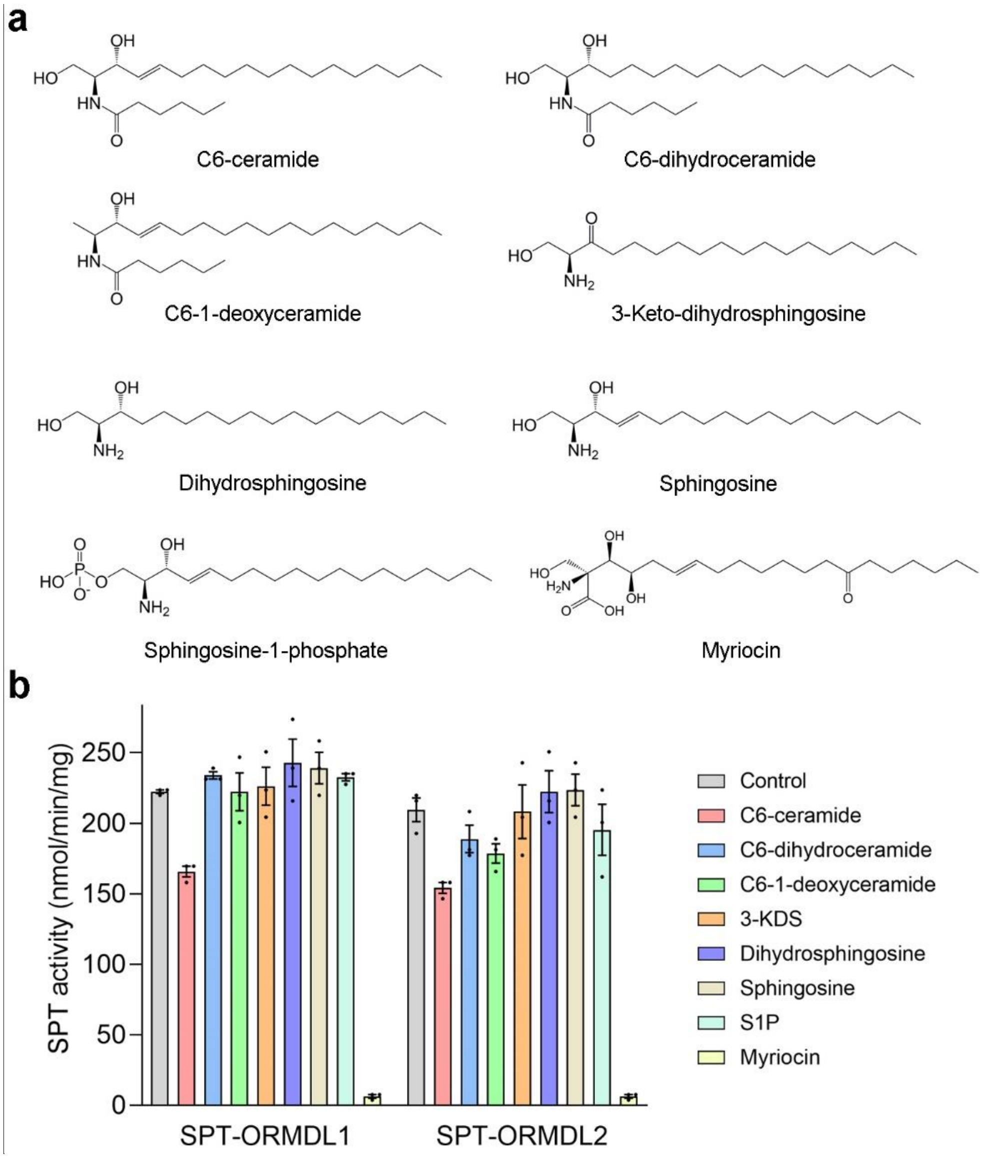

Supplementary Fig. 2 | Selective inhibition of SPT-ORMDL1 and SPT-ORMDL2 by C6-ceramide. **a,** Chemical structure of C6-ceramide and the other sphingolipid species or analogs used in this study. **b,** Selective inhibition of SPT-ORMDL1 and SPT-ORMDL2 by C6-ceramide among various sphingolipid species or analogs. The different sphingolipid species or analogs were applied at 10 μM in each assay. Data are presented as mean values ± SEM of three independent experiments. Source data are provided as a Source Data file.

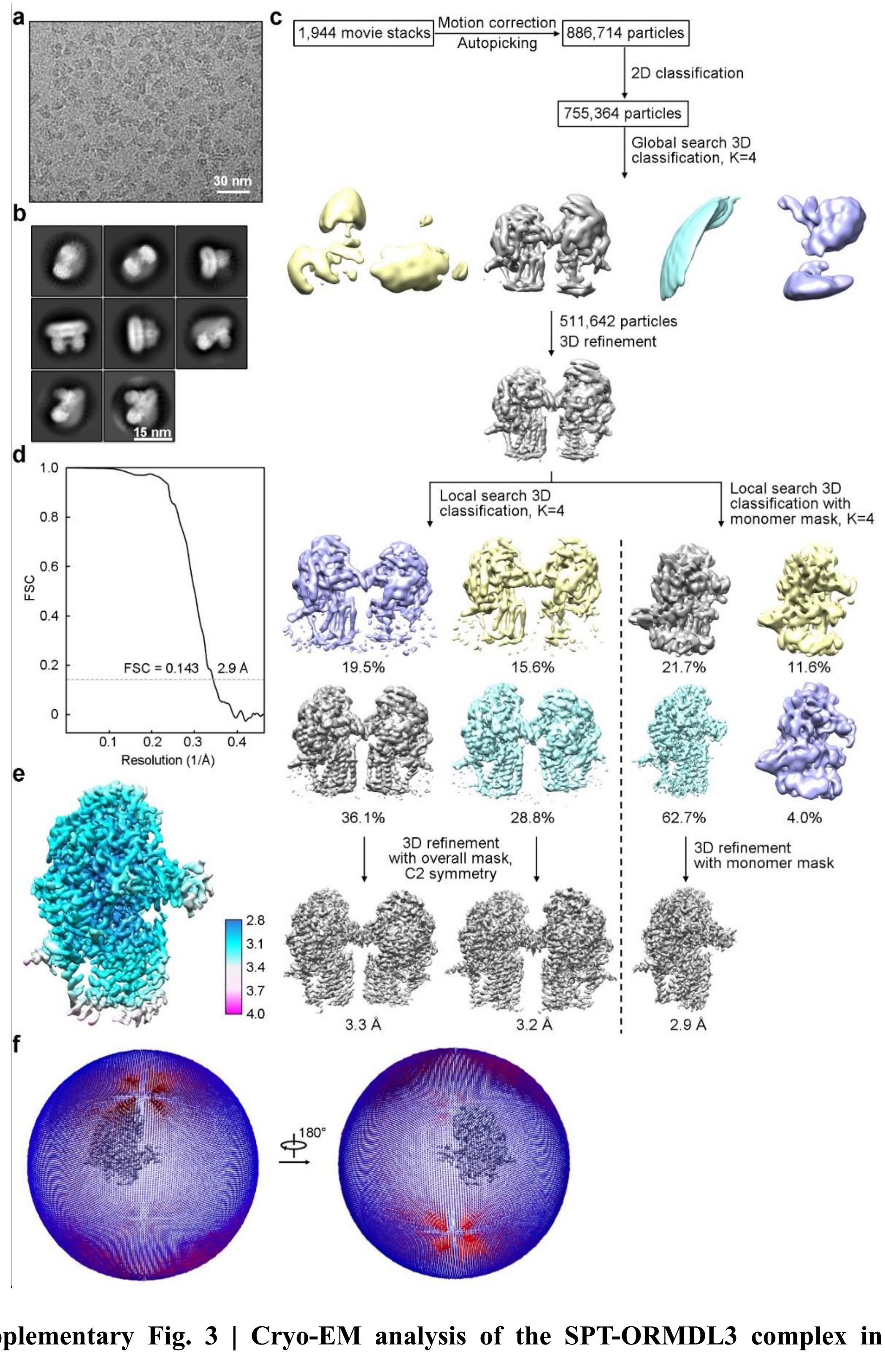

Supplementary Fig. 3 | Cryo-EM analysis of the SPT-ORMDL3 complex in the presence of excess C6-ceramide. **a,** Representative cryo-EM micrograph. **b,**

Representative 2D class averages. **c,** Flowchart for cryo-EM data processing. **d,** Gold-standard FSC curve for the map of the monomeric complex generated using Relion 3.0. **e,** Local resolution map of the monomeric complex. The color code for resolutions, shown with the unit Å, is calculated using Relion 3.0. **f,** Angular distribution of particles in the 3D reconstruction.

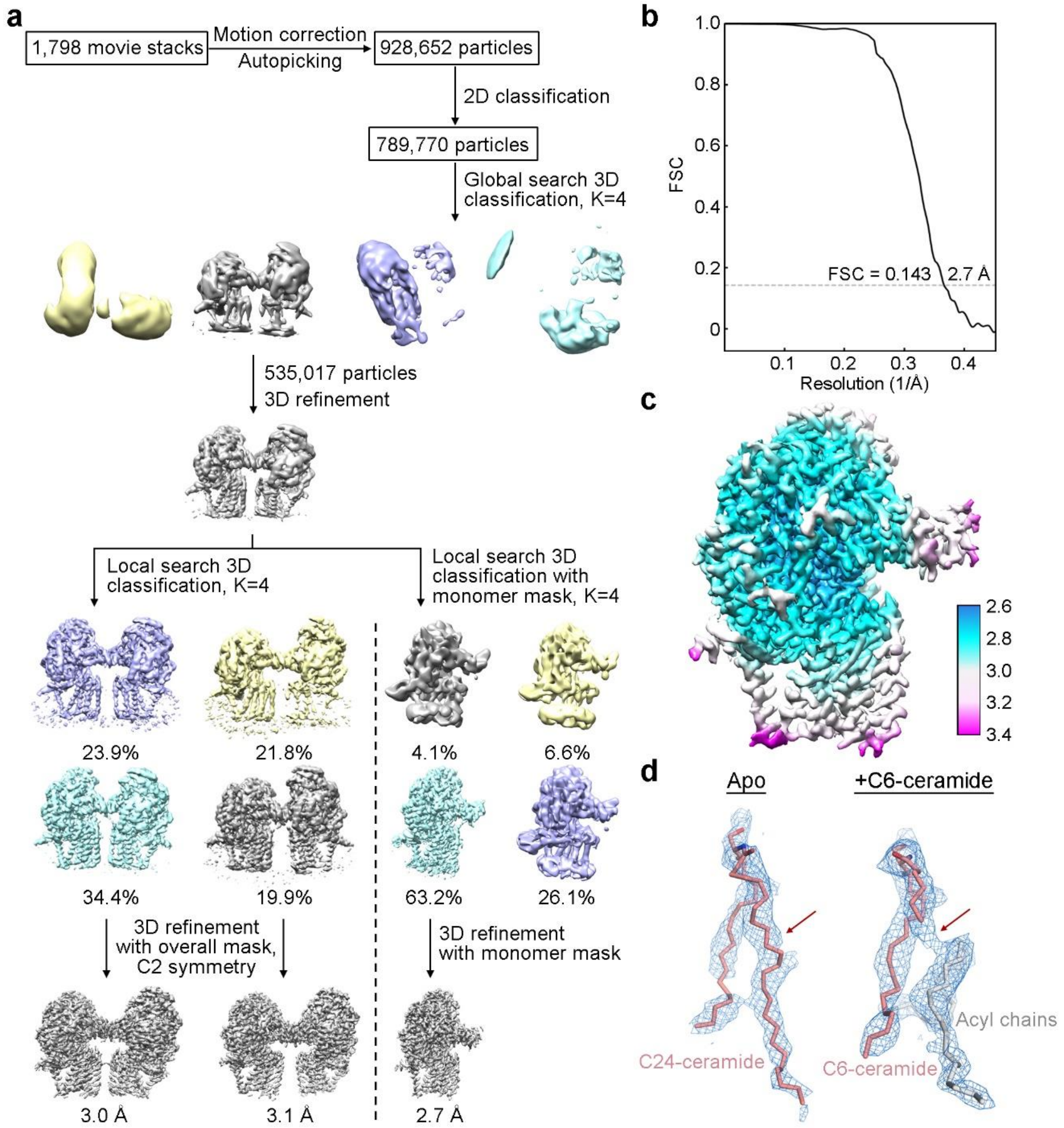

Supplementary Fig. 4 | Cryo-EM analysis of the apo SPT-ORMDL3 complex. **a,** Flowchart for cryo-EM data processing. **b,** Gold-standard FSC curve for the monomeric apo SPT-ORMDL3 map generated using Relion 3.0. **c,** Local resolution map of the monomeric apo SPT-ORMDL3. The color code for resolutions, shown with the unit Å, is calculated using Relion 3.0. **d,** Comparison of the EM maps for the lipid-like densities between SPTLC2 and ORMDL3 in the apo SPT-ORMDL3 complex and C6-ceramide-

bound SPT-ORMDL3 complex. The densities, shown in blue meshes, were contoured at 5σ. The density for the amide-linked fatty acyl chain of ceramide from the apo SPT-ORMDL3 map is more continuous than that from the C6-ceramide-bound SPT-ORMDL3 map, thus better accommodating a C24-ceramide molecule. The ceramide molecules and potential acyl chains are shown as pink and gray sticks, respectively.

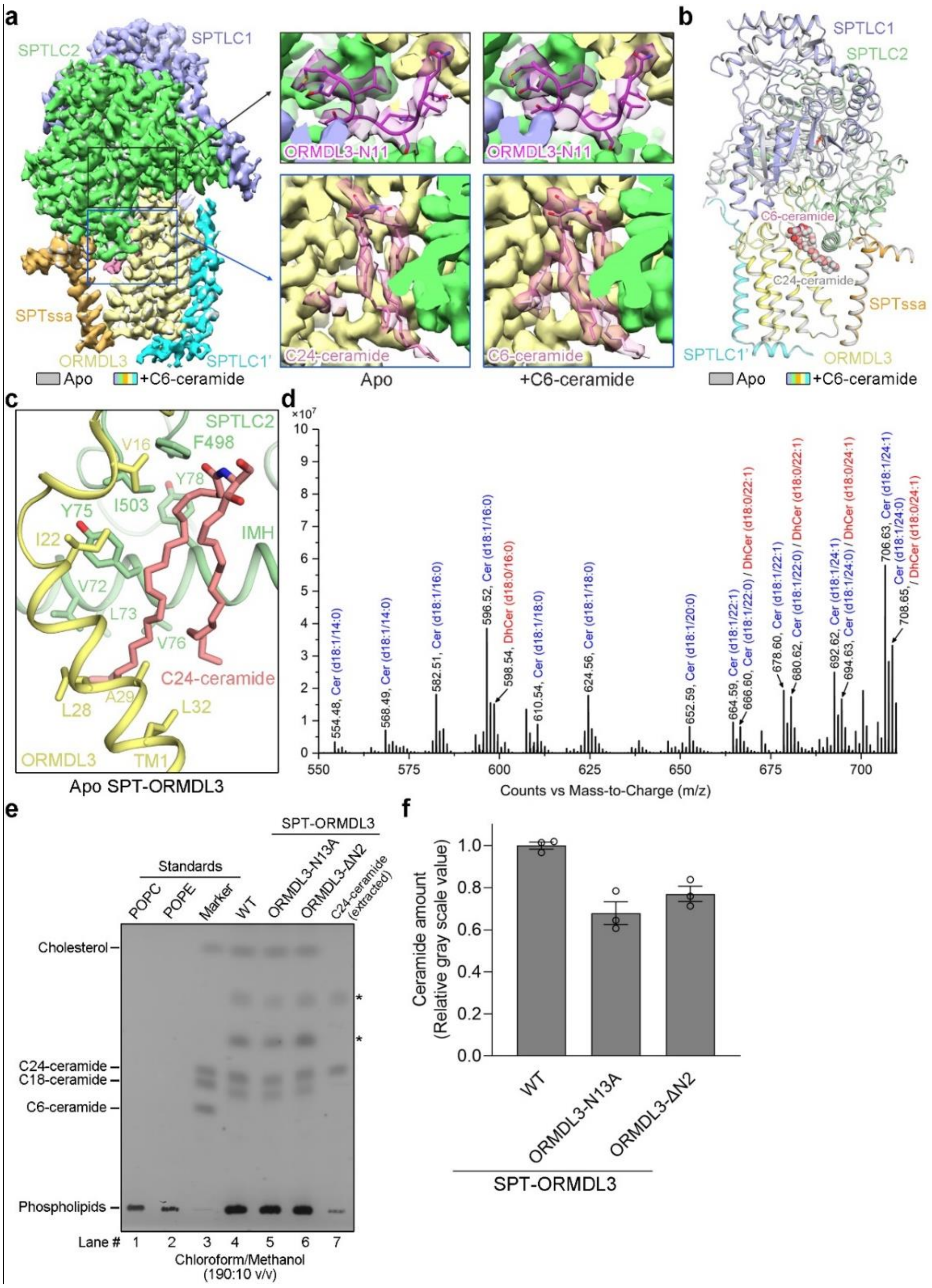

Supplementary Fig. 5 | Comparison of the apo SPT-ORMDL3 complex and C6-ceramide-bound SPT-ORMDL3 complex and characterization of the endogenously bound ceramides. **a,** Comparison of the EM maps of apo SPT-ORMDL3 complex and C6-ceramide-bound SPT-ORMDL3 complex. The apo SPT-ORMDL3 complex is colored

gray. The C6-ceramide-bound SPT-ORMDL3 complex is colored based on the subunits. Insets: The maps for ORMDL3-N11 and ceramide from the apo SPT-ORMDL3 complex are similar to those from the C6-ceramide-bound SPT-ORMDL3 complex. **b,** Superposition of the structural models of apo SPT-ORMDL3 complex and C6-ceramide-bound SPT-ORMDL3 complex. C6-ceramide and C24-ceramide are shown as pink and gray spheres, respectively. **c,** A detailed view of the hydrophobic interactions between C24-ceramide acyl-chain and SPT-ORMDL3. **d,** Mass spectrometry analysis of endogenous ceramides co-purified with SPT-ORMDL3. The major ceramide (Cer) and dihydroceramide (DhCer) species were highlighted in the mass spectra data. All the ions were confirmed by tandem MS spectra. **e,** Visualization and quantification of endogenous ceramides co-purified with WT SPT-ORMDL3, ORMDL3-N13A mutant, and ORMDL3-ΔN2 mutant by TLC. Calculation of the ceramide levels extracted from 0.3 mg protein complexes (lanes #4-6) was based on that extracted from 1.33 μg C24-ceramide standard (lane #7). It was determined that the amounts of extracted ceramide (C18~C24 ceramides) from 0.3 mg samples of WT SPT-ORMDL3, ORMDL3-N13A mutant, and ORMDL3-ΔN2 mutant were roughly 3.8 μg, 2.5 μg, and 2.89 μg, respectively. The molar stoichiometry between WT SPT-ORMDL3 protomer or SPT-ORMDL3 (ORMDL3-N13A) protomer or SPT-ORMDL3 (ORMDL3-ΔN2) protomer and bound ceramide was estimated to be 1:2.94 or 1:1.94 or 1:2.22, respectively. The asterisks mark unidentified components. **f,** The amounts of co-purified ceramide from the ORMDL3-N13A mutant and ORMDL3-ΔN2 mutant were normalized to that from the WT SPT-ORMDL3 complex. The results represent the average ± SEM of three independent experiments. Source data are provided as a Source Data file.

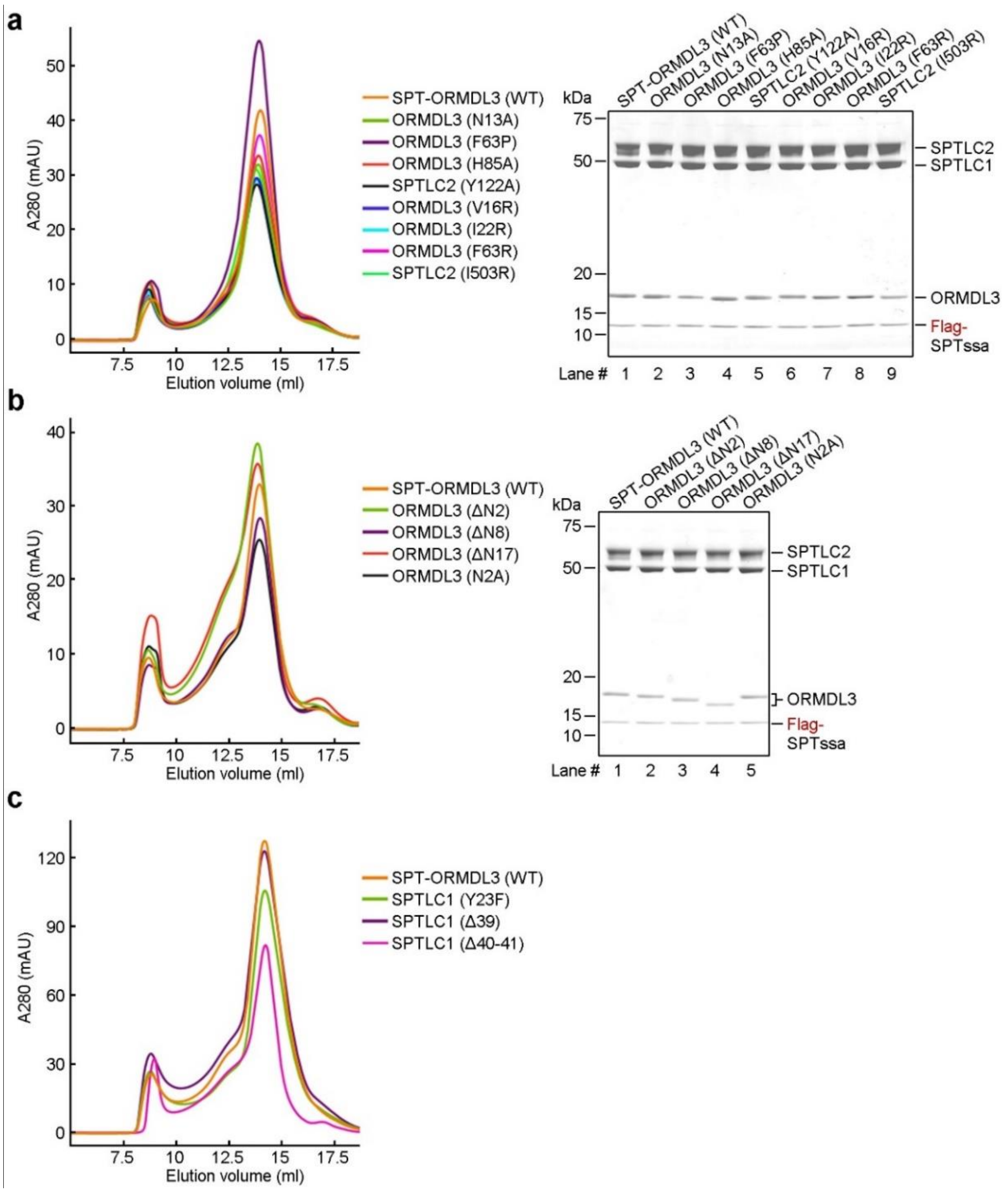

Supplementary Fig. 6 | Size exclusion chromatography (SEC) profiles of SPT-ORMDL3 variants. **a-b,** SEC profiles of the ceramide-binding variants (**a**) and the ORMDL3 N-terminal deletion and N2A variants (**b**). The purified protein was visualized by Coomassie-blue stained SDS-PAGE gel. **c,** SEC profiles of the ALS-associated SPTLC1 variants. AU represents arbitrary units. Source data are provided as a Source Data file.

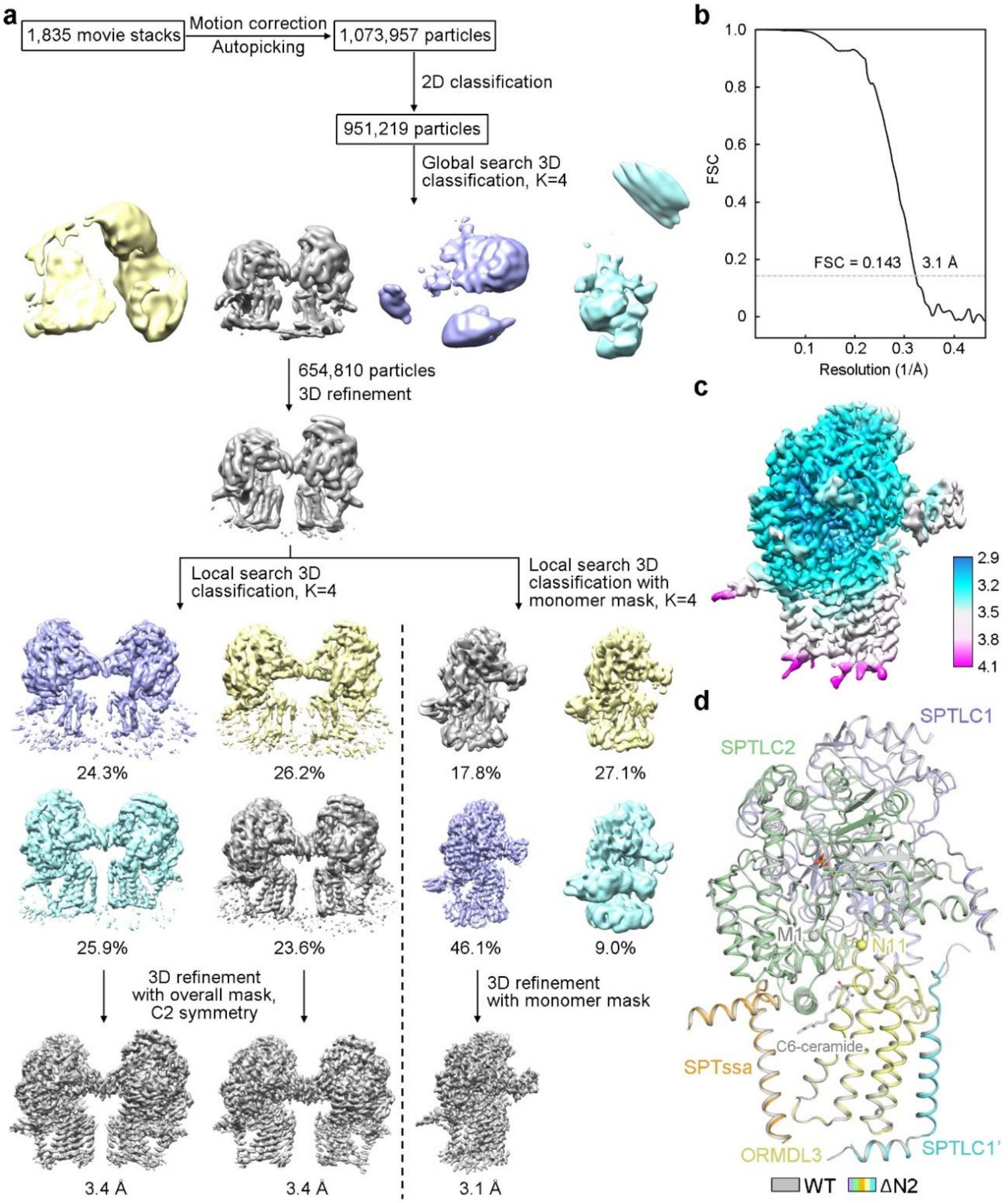

Supplementary Fig. 7 | Cryo-EM analysis of the SPT-ORMDL3 (ORMDL3-ΔN2) complex. **a,** Flowchart for cryo-EM data processing. **b,** Gold-standard FSC curve for the monomeric ORMDL3-ΔN2 mutant map generated using Relion 3.0. **c,** Local resolution map of the monomeric ORMDL3-ΔN2 mutant. The color code for resolutions,

shown with the unit Å, is calculated using Relion 3.0. **d,** Superposition of the ORMDL3-ΔN2 mutant and C6-ceramide-bound SPT-ORMDL3 complex. C6-ceramide-bound SPT-ORMDL3 complex is colored gray. The ORMDL3-ΔN2 mutant is colored based on the subunits. Met1 and Asn11, the resolved N-terminus of WT ORMDL3 and ORMDL3 -ΔN2, are shown as gray and yellow spheres, respectively. C6-ceramide is shown as gray sticks.

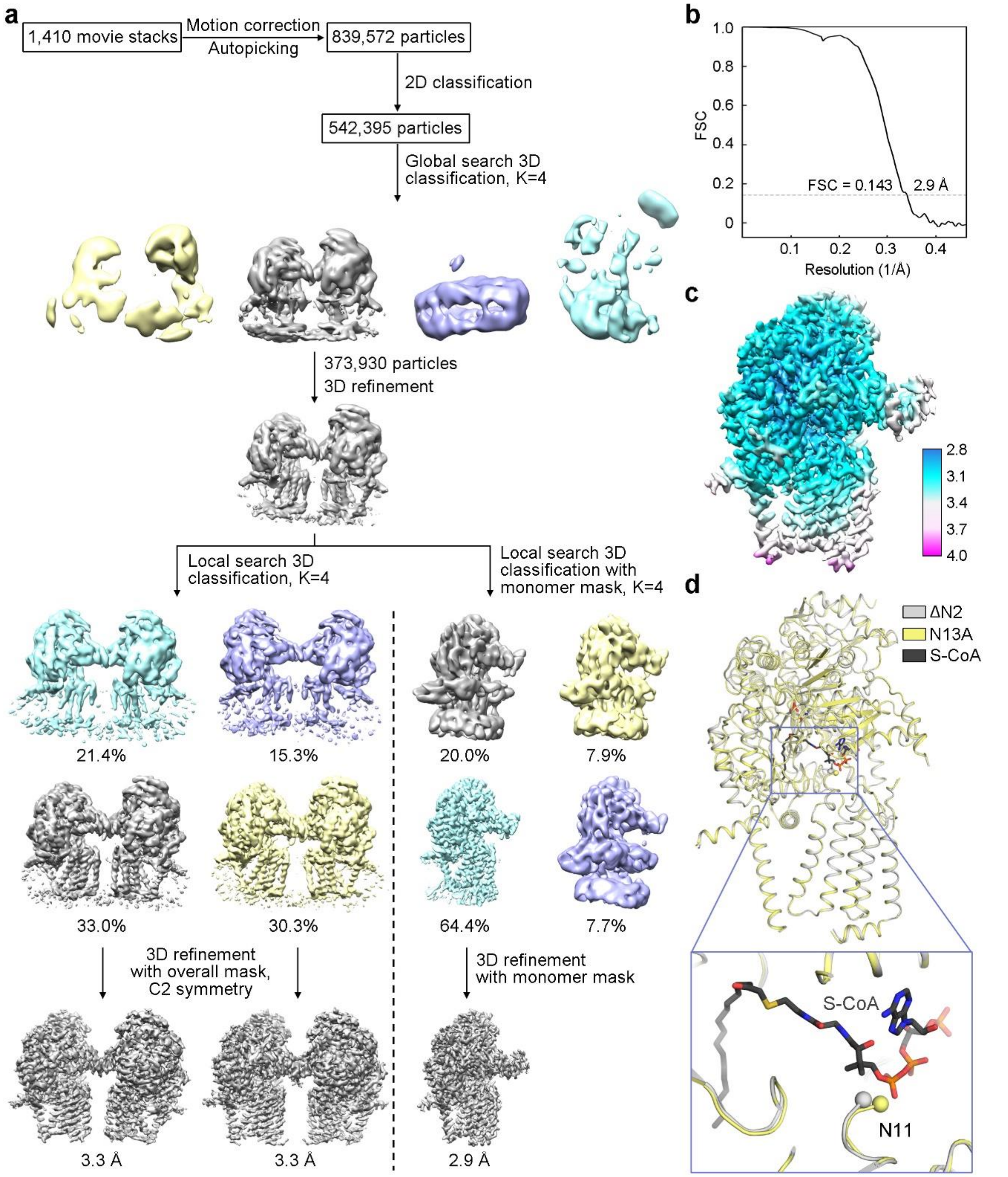

Supplementary Fig. 8 | Cryo-EM analysis of the SPT-ORMDL3 (ORMDL3-N13A) complex.

a, Flowchart for cryo-EM data processing. **b,** Gold-standard FSC curve for the ORMDL3-N13A mutant map generated using Relion 3.0. **c,** Local resolution map of the ORMDL3-N13A mutant. The color code for resolutions, shown with the unit Å, is

calculated using Relion 3.0. **d,** Superposition of the ORMDL3-ΔN2 mutant structure, the ORMDL3-N13A mutant structure, and the S-CoA-bound SPT-ORMDL3 structure (PDB 7CQK). The ORMDL3-ΔN2 mutant structure is colored gray. The ORMDL3-N13A mutant structure is colored yellow. S-CoA is displayed as black sticks. The resolved N-terminus of ORMDL3-ΔN2 and ORMDL3-N13A are both Asn11, shown as gray and yellow spheres, respectively.

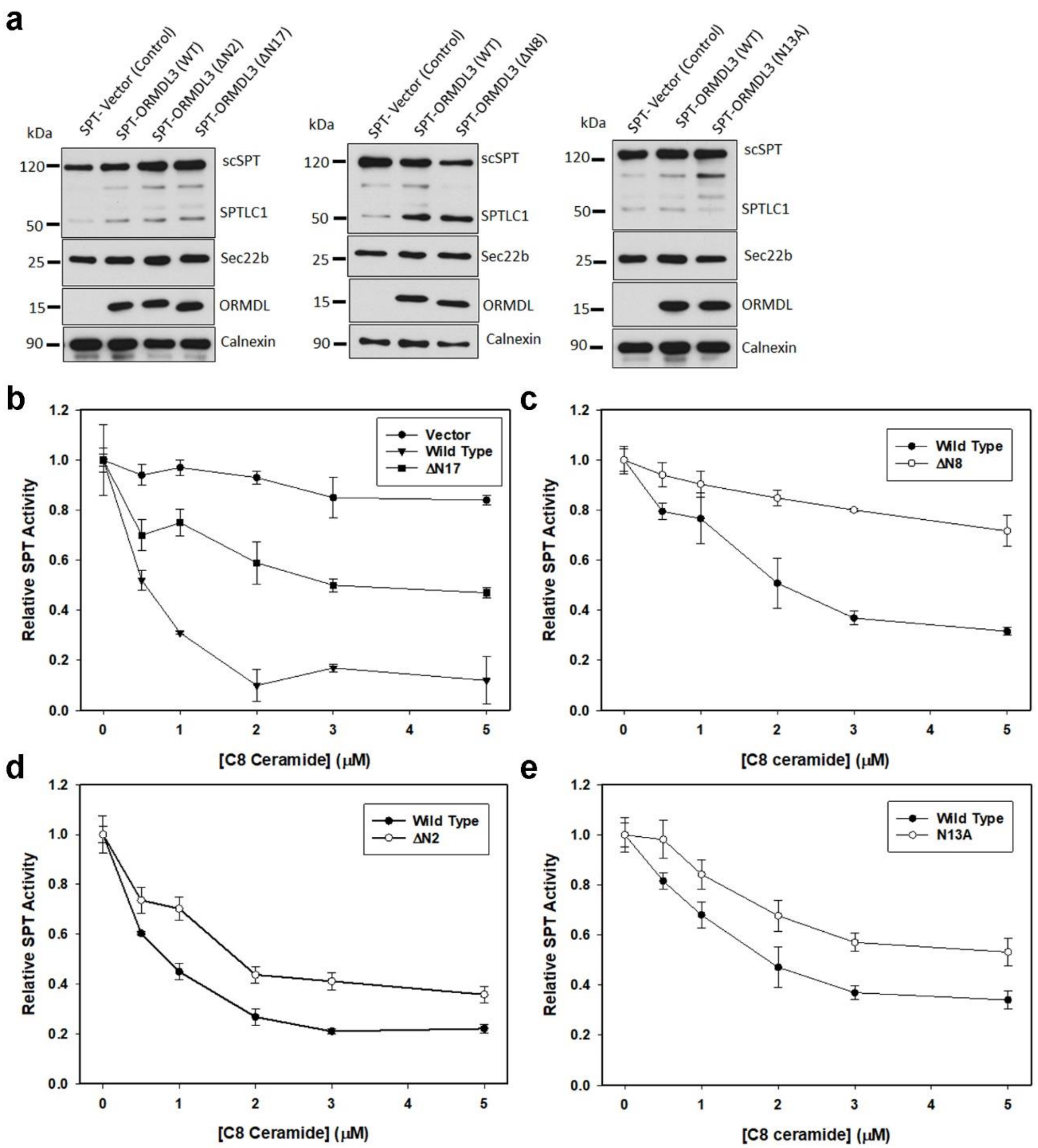

Supplementary Fig. 9 | Mutations of ORMDL3 in the N-terminus and ceramide binding site blunt the response of the SPT complex to ceramide in native membranes. **a.** Mutant constructs of ORMDL3 are expressed at similar levels in membranes used to test inhibitory activity. Membranes were prepared from cells in which the endogenous ORMDLs had been depleted by Crispr/Cas9 gene editing and siRNA transfection and in which co-translational assembly of SPT/ORMDL complex was accomplished by

simultaneous transfection of the ORMDL construct to be tested and a fusion protein consisting of SPTLC1, SPTLC2, and SPTssa. Immunoblotting was performed to assess the level of expression of single-chain SPT (scSPT), SPTLC1, WT human ORMDL3 and human ORMDL3 mutant constructs for the samples assayed in panels **b**-**e**. 0.5 μg of total membrane protein was used per lane and two separate gels were run for the detection of SPT and ORMDL3. Sec22b, a membrane protein is used as a loading control for scSPT/SPTLC1 immunoblotting, and Calnexin, an ER chaperon is used as a loading control for ORMDL immunoblotting. **b**-**e**, SPT activity in response to C8-ceramide levels was measured in membrane fractions derived from cells expressing ΔN17 (**b**), ΔN8 (**c**), ΔN2 (**d**), or N13A (**e**). A WT control was included in each assay, the vector control is shown in **b**. Each point reflects the mean of quadruplicate technical replicates, error bars indicate standard deviation. Each panel is representative of at least three replicate assays. Source data are provided as a Source Data file.

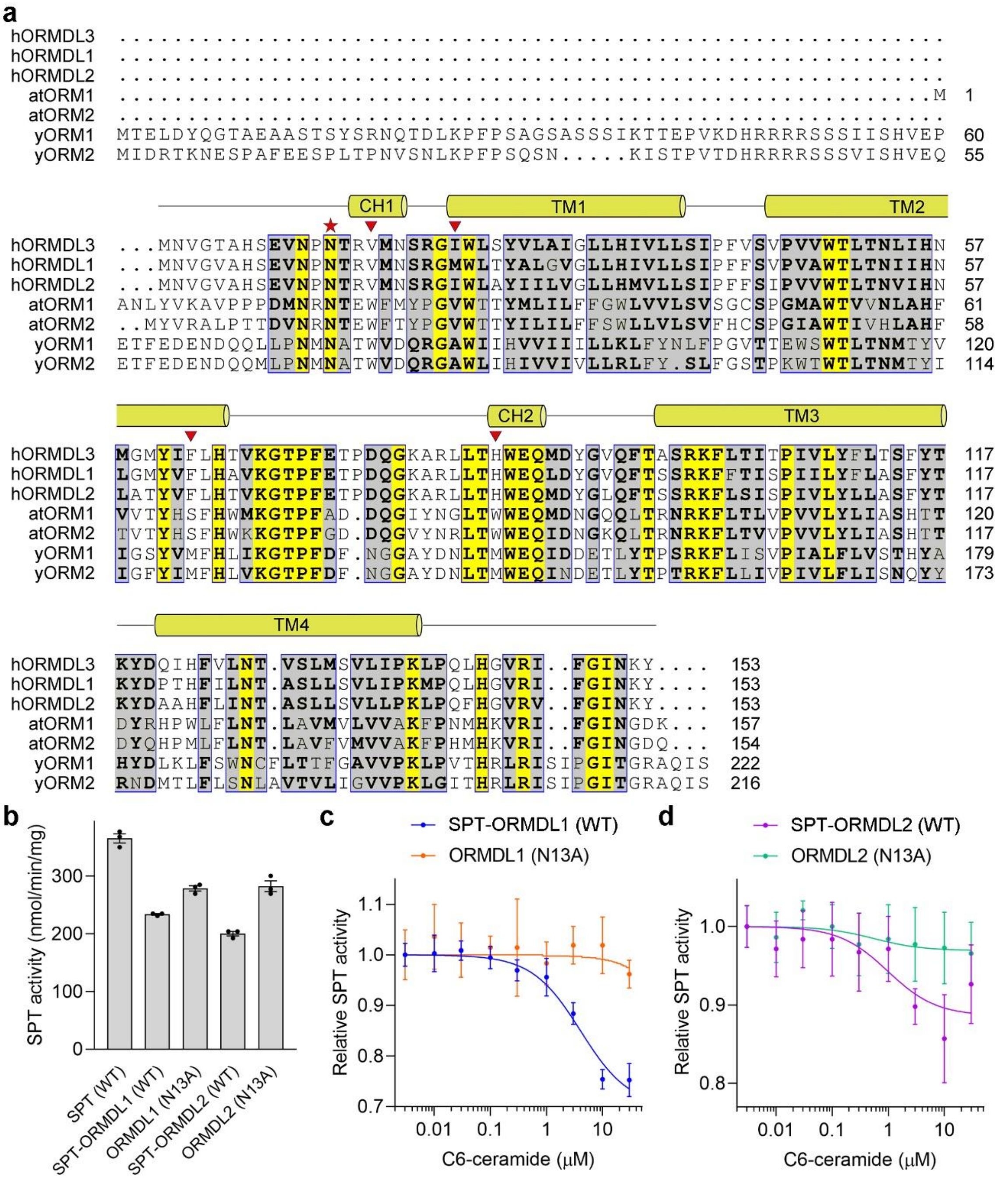

Supplementary Fig. 10 | Sequence alignments of ORMDLs/ORMs and SPT activity of the SPT-ORMDL1 (ORMDL1-N13A) and SPT-ORMDL2 (ORMDL2-N13A) variants. **a,** Sequence alignment of ORMDLs/ORMs sequences from different species. Secondary structural elements of human ORMDL3 are labeled above the sequence

alignment. CH: cytosolic helix. TM: transmembrane helix. The red star indicates the invariant asparagine residue crucial for ceramide binding. The red triangles indicate the other residues involved in ceramide coordination in human ORMDL3. The UniProt IDs for the aligned sequences are as follows: hORMDL3: Q8N138; hORMDL1: Q9P0S3; hORMDL2: Q53FV1; atORM1: Q9C5I0; atORM2: Q9FHY3; yORM1: P53224; and yORM2: Q06144. “h” for *Homo sapiens* (Human), “at” for *Arabidopsis thaliana*, and “y” for *Saccharomyces cerevisiae* (yeast). **b,** SPT activity of the ORMDL1-N13A and ORMDL2-N13A mutants. Data are presented as mean values ± SEM of three independent experiments. **c-d,** The great loss of C6-ceramide-mediated inhibition of the ORMDL1-N13A mutant (**c**) and the ORMDL2-N13A mutant (**d**). Data are presented as mean values ± SEM of three independent experiments. The IC_{50} values were summarized in Supplementary Table 1. Source data are provided as a Source Data file.

Supplementary Table 1 | IC_{50} values of C6-ceramide for SPT-ORMDL variants.

Batch	SPT-ORMDL variants	IC_{50} (μM) *
1	WT SPT-ORMDL3	2.151 (1.632 – 2.829)
	WT SPT-ORMDL1	4.067 (2.006 – 8.262)
	ORMDL1-N13A	N/A
	WT SPT-ORMDL2	0.9802 (0.02669 – 8.047)
	ORMDL2-N13A	N/A
2	WT SPT-ORMDL3	1.522 (0.9947 – 2.310)
	ORMDL3-N13A	N/A
	ORMDL3-F63P	N/A
	ORMDL3-H85A	0.9638 (0.6555 – 1.396)
	SPTLC2-Y122A	6.984 (3.866 – 13.08)
3	WT SPT-ORMDL3	1.560 (1.031 – 2.321)
	ORMDL3-V16R	N/A
	ORMDL3-I22R	N/A
	ORMDL3-F63R	N/A
	SPTLC2-I503R	N/A
4	WT SPT-ORMDL3	3.297 (2.662 – 4.084)
	ORMDL3-ΔN2	N/A
	ORMDL3-ΔN8	N/A
	ORMDL3-ΔN17	N/A
	ORMDL3-N2A	N/A
5	WT SPT-ORMDL3	1.353 (0.860 – 2.097)
	SPTLC1-Y23F	1.442 (0.886 – 2.294)
	SPTLC1-Δ39	2.200 (0.410 – 8.513)
	SPTLC1-Δ40-41	N/A

* IC_{50} values obtained by fitting normalized activity data shown in Figs. 1, 3, 4, 6, and Supplementary Fig. 10 (with a 95% confidence interval). N/A = not available.

Supplementary Table 2 | Data collection, refinement and validation statistics.

	SPT-ORMDL3 + C6-ceramide (EMD-33864, PDB 7YIU)	Apo SPT-ORMDL3 (EMD-33866, PDB 7YIY)	SPT-ORMDL3 (ORMDL3-ΔN2) (EMD-33868, PDB 7YJ1)	SPT-ORMDL3 (ORMDL3-N13A) (EMD-33869, PDB 7YJ2)
**Data collection and processing**				
Magnification	130,000	130,000	130,000	130,000
Voltage (kV)	300	300	300	300
Electron exposure ($e^{-}/Å^2$)	50	50	50	50
Defocus range (μm)	-2.0 to -1.0	-2.0 to -1.0	-2.0 to -1.0	-2.0 to -1.0
Pixel size (Å)	1.08	1.08	1.08	1.08
Symmetry imposed	C1	C1	C1	C1
Initial particle images (no.)	886,714	928,652	1,073,957	839,572
Final particle images (no.)	337,730	358,765	317,358	249,589
Map resolution (Å)	2.9 Å	2.7 Å	3.1 Å	2.9 Å
FSC threshold	0.143	0.143	0.143	0.143
Map resolution range (Å)	2.8-4.0 Å	2.6-3.4 Å	2.9-4.1 Å	2.8-4.0 Å
**Refinement**				
Initial model used (PDB code)	6M4O	6M4O	6M4O	6M4O
Model resolution (Å)	2.9 Å	2.7 Å	3.1 Å	2.9 Å
FSC threshold	0.143	0.143	0.143	0.143
Model resolution range (Å)				
Map sharpening *B* factor ($Å^2$)	-100	-84	-104	-101
Model composition				
Nonhydrogen atoms	9,285	9,257	9,140	9,124
Protein residues	1,166	1,164	1,154	1,154
Ligands	4	2	1	1
B factors ($Å^2$)				
Protein	38.49	39.27	53.05	46.65
Ligand	33.58	36.20	40.38	32.06
R.m.s. deviations				
Bond lengths (Å)	0.005	0.007	0.008	0.007
Bond angles (°)	0.779	0.910	0.772	0.780
**Validation**				
MolProbity score	1.67	1.58	1.74	1.63
Clashscore	4.66	3.94	5.45	4.53
Poor rotamers (%)	0.00	0.00	0.00	0.00
Ramachandran plot				
Favored (%)	93.34	94.02	92.99	93.96
Allowed (%)	6.66	5.89	6.92	6.04
Disallowed (%)	0.00	0.09	0.09	0.00

Supplementary Table 3 | Primers used in the study.

Primer	Sequence (5’-3’)
SPTLC1-pCAG-1-KpnI-F	CGGGGTACCATGGCTACCGCTACCGAG
SPTLC1-pCAG-473-XhoI-R	CCGCTCGAGTTACAGCAGCACGGCCTG
SPTLC1 (Y23F)-F	TACGAAGCTCCTGCCTTCCATCTTATCCTGGAG
SPTLC1 (Y23F)-R	CTCCAGGATAAGATGGAAGGCAGGAGCTTCGTA
SPTLC1 (Δ39)-F	CTGTGGATCATCAGACTGTTCAGCAAGACCTACAAG
SPTLC1 (Δ39)-R	CTTGTAGGTCTTGCTGAACAGTCTGATGATCCACAG
SPTLC1 (Δ40-41)-F	TGGATCATCAGACTGCTGAAGACCTACAAGCTGCAG
SPTLC1 (Δ40-41)-R	CTGCAGCTTGTAGGTCTTCAGCAGTCTGATGATCCA
SPTLC2-pCAG-1-KpnI-F	CGGGGTACCATGAGGCCCGAACCTGGA
SPTLC2-pCAG-562-XhoI-R	CCGCTCGAGTTAGTCCTCGGTTTCTTC
SPTLC2 (Y122A)-F	GACTTCGAGAACTTCGCTACCAGAAACCTGTAC
SPTLC2 (Y122A)-R	GTACAGGTTTCTGGTAGCGAAGTTCTCGAAGTC
SPTLC2 (I503R)-F	TTTCCTGCCACCCCTCGCATTGAGAGCAGAGCC
SPTLC2 (I503R)-R	GGCTCTGCTCTCAATGCGAGGGGTGGCAGGAAA
SPTssa-pCAG-1-NotI-F	ATTTGCGGCCGCATGGCTGGAATGGCTCTG
SPTssa-pCAG-71-XhoI-R	CCGCTCGAGTCACTGGACGATCTCGAA
ORMDL1-pCAG-1-KpnI-F	CGGGGTACCATGAATGTGGGAGTGGCC
ORMDL1-pCAG-153-XhoI-R	CCGCTCGAGTCAGTACTTGTTGATGCC
ORMDL1 (N13A)-F1	GCCCACTCTGAAGTGAATCCCGCCACCAGAGTGATGAAC
ORMDL1 (N13A)-F2	CGGGGTACCATGAATGTGGGAGTGGCCCACTCTGAAGTG
ORMDL2-pCAG-1-KpnI-F	CGGGGTACCATGAATGTGGGCGTGGCC
ORMDL2-pCAG-153-XhoI-R	CCGCTCGAGTCAGTACTTGTTGATGCC
ORMDL2 (N13A)-F1	GCCCATTCTGAAGTGAACCCCGCCACCAGAGTGATGAAC
ORMDL2 (N13A)-F2	CGGGGTACCATGAATGTGGGCGTGGCCCATTCTGAAGTG
ORMDL3-pCAG-1-KpnI-F	CGGGGTACCATGAATGTGGGAACCGCC
ORMDL3-pCAG-153-XhoI-R	CCGCTCGAGTCAGTACTTGTTGATGCC
ORMDL3 (N2A)-pCAG-1-KpnI-F	CGGGGTACCATGGCTGTGGGAACCGCCCAC
ORMDL3 (N13A)-pCAG-1-KpnI-F	CGGGGTACCATGAATGTGGGAACCGCCCACAG<br>CGAGGTTAATCCCGCCACCAGAGTGATGAAC
ORMDL3 (V16R)-F	AATCCCAACACCAGACGCATGAACAGCAGAGGC
ORMDL3 (V16R)-R	GCCTCTGCTGTTCATGCGTCTGGTGTTGGGATT
ORMDL3 (I22R)-F	ATGAACAGCAGAGGCCGCTGGCTGAGCTATGTG
ORMDL3 (I22R)-R	CACATAGCTCAGCCAGCGGCCTCTGCTGTTCAT
ORMDL3 (F63P)-F	ATGGGCATGTACATCCCTCTGCACACCGTGAAG
ORMDL3 (F63P)-R	CTTCACGGTGTGCAGAGGGATGTACATGCCCAT
ORMDL3 (F63R)-F	ATGGGCATGTACATCCGCCTGCACACCGTGAAG
ORMDL3 (F63R)-R	CTTCACGGTGTGCAGGCGGATGTACATGCCCAT
ORMDL3 (H85A)-F	GCTAGACTGCTGACCGCCTGGGAGCAAATG
ORMDL3 (H85A)-R	CATTTGCTCCCAGGCGGTCAGCAGTCTAGC
ORMDL3 (ΔN2)-pCAG-3-KpnI-F	CGGGGTACCATGGTGGGAACCGCCCACAGC
ORMDL3 (ΔN8)-pCAG-9-KpnI-F	CGGGGTACCATGGAGGTTAATCCCAACACC
ORMDL3 (ΔN17)-pCAG-18-KpnI-F	CGGGGTACCATGAACAGCAGAGGCATCTGG
Vector-pCAG-F	GCAACGTGCTGGTTATTGTG
