# Supplementary material for: Ceramide sensing by human SPT-ORMDL complex for establishing sphingolipid homeostasis
Source: Nat Commun. 2023 Jun 13;14:3475. doi: 10.1038/s41467-023-39274-y (PMC10261145; doi:10.1038/s41467-023-39274-y)
Supplement: Supplementary file 3 — Reporting Summary [file 41467_2023_39274_MOESM3_ESM.pdf]

nature portfolio

Corresponding author(s): Binks W. Wattenberg, Xin Gong

Last updated by author(s): May 18, 2023

Reporting Summary

Nature Portfolio wishes to improve the reproducibility of the work that we publish. This form provides structure for consistency and transparency in reporting. For further information on Nature Portfolio policies, see our Editorial Policies and the Editorial Policy Checklist.

Statistics

For all statistical analyses, confirm that the following items are present in the figure legend, table legend, main text, or Methods section.

n/a	Confirmed	
☐	☒	The exact sample size (*n*) for each experimental group/condition, given as a discrete number and unit of measurement
☐	☒	A statement on whether measurements were taken from distinct samples or whether the same sample was measured repeatedly
☒	☐	The statistical test(s) used AND whether they are one- or two-sided<br>*Only common tests should be described solely by name; describe more complex techniques in the Methods section.*
☒	☐	A description of all covariates tested
☒	☐	A description of any assumptions or corrections, such as tests of normality and adjustment for multiple comparisons
☐	☒	A full description of the statistical parameters including central tendency (e.g. means) or other basic estimates (e.g. regression coefficient) AND variation (e.g. standard deviation) or associated estimates of uncertainty (e.g. confidence intervals)
☒	☐	For null hypothesis testing, the test statistic (e.g. *F*, *t*, *r*) with confidence intervals, effect sizes, degrees of freedom and *P* value noted<br>*Give P values as exact values whenever suitable.*
☒	☐	For Bayesian analysis, information on the choice of priors and Markov chain Monte Carlo settings
☒	☐	For hierarchical and complex designs, identification of the appropriate level for tests and full reporting of outcomes
☒	☐	Estimates of effect sizes (e.g. Cohen's *d*, Pearson's *r*), indicating how they were calculated

Our web collection on statistics for biologists contains articles on many of the points above.

Software and code

Policy information about availability of computer code

Data collection	SerialEM 3.7.0
Data analysis	MotionCor2 1.0.5, Gctf 1.06, RELION 3.0, Coot 0.8.9, PHENIX 1.13, Pymol 2.0.7, Chimera 1.13, MolProbity

For manuscripts utilizing custom algorithms or software that are central to the research but not yet described in published literature, software must be made available to editors and reviewers. We strongly encourage code deposition in a community repository (e.g. GitHub). See the Nature Portfolio guidelines for submitting code & software for further information.

Data

Policy information about availability of data

All manuscripts must include a data availability statement. This statement should provide the following information, where applicable:

- Accession codes, unique identifiers, or web links for publicly available datasets
- A description of any restrictions on data availability
- For clinical datasets or third party data, please ensure that the statement adheres to our policy

The EM density maps generated in this study have been deposited in the EMDB under accession codes EMD-33864 [https://www.ebi.ac.uk/emdb/EMD-33864] (SPT-ORMDL3 + C6-ceramide), EMD-33866 [https://www.ebi.ac.uk/emdb/EMD-33866] (apo SPT-ORMDL3), EMD-33868 [https://www.ebi.ac.uk/emdb/EMD-33868] (ORMDL3-ΔN2 variant), and EMD-33869 [https://www.ebi.ac.uk/emdb/EMD-33869] (ORMDL3-N13A variant). The atomic coordinates have been deposited in the PDB under the accession codes 7YIU [https://doi.org/10.2210/pdb7YIU/pdb] (SPT-ORMDL3 + C6-ceramide), 7YIY [https://doi.org/10.2210/pdb7YIY/pdb] (apo SPT-

ORMDL3), 7YJ1 [https://doi.org/10.2210/pdb7YJ1/pdb] (ORMDL3-ΔN2 variant), and 7YJ2 [https://doi.org/10.2210/pdb7YJ2/pdb] (ORMDL3-N13A variant). Source data are provided with this paper.

Human research participants

Policy information about studies involving human research participants and Sex and Gender in Research.

Reporting on sex and gender	N/A
Population characteristics	N/A
Recruitment	N/A
Ethics oversight	N/A

Note that full information on the approval of the study protocol must also be provided in the manuscript.

Field-specific reporting

Please select the one below that is the best fit for your research. If you are not sure, read the appropriate sections before making your selection.

☒ Life sciences ☐ Behavioural & social sciences ☐ Ecological, evolutionary & environmental sciences

For a reference copy of the document with all sections, see nature.com/documents/nr-reporting-summary-flat.pdf

Life sciences study design

All studies must disclose on these points even when the disclosure is negative.

Sample size	No statistical method was used to determine sample size; sample sizes were chosen based on the standard in the field.
Data exclusions	No data was excluded.
Replication	Each experiment was reproduced at least three times on separate occasions. Experimental findings were reliably reproduced.
Randomization	Samples were not randomized because these studies involved defined molecular reagents used in specific mechanistic assays. Randomization is not standard in this field. This study did not involve animals or human research participants.
Blinding	Blinding was not necessary because experimental conditions were well-controlled and experimental results were quantitative and did not require subjective interpretation or analysis. Blinding is not standard in this field. This study did not involve animals or human research participants.

Reporting for specific materials, systems and methods

We require information from authors about some types of materials, experimental systems and methods used in many studies. Here, indicate whether each material, system or method listed is relevant to your study. If you are not sure if a list item applies to your research, read the appropriate section before selecting a response.

Materials & experimental systems

n/a	Involved in the study	
☐	☒	Antibodies
☐	☒	Eukaryotic cell lines
☒	☐	Palaeontology and archaeology
☒	☐	Animals and other organisms
☒	☐	Clinical data
☒	☐	Dual use research of concern

Methods

n/a	Involved in the study	
☒	☐	ChIP-seq
☒	☐	Flow cytometry
☒	☐	MRI-based neuroimaging

Antibodies

Antibodies used	Mouse monoclonal anti-SPTLC1-Beckton Dickinson Cat # 611305 Lot #8093914.<br>Mouse monoclonal anti-Sec22b Santa Cruz Cat # Sc-101267 Lot #E1022.

	Rabbit polyclonal anti-ORMDL Millipore Cat. #ABN417 Lot # 3738434.<br>Rabbit polyclonal anti-Calnexin Enzo Life Sciences Cat. #-ADI-SPA-860-F Lot #08052188.
Validation	anti-SPTLC1: Validation-This laboratory by overexpression in Hela cells.<br>anti-Sec22b: Validation, by manufacturer by overexpression in Hela cells.<br>anti-ORMDL: Validation by this laboratory by overexpression, and knockdown by siRNA and Crispr/Cas9 in Hela cells.<br>anti-Calnexin: Validation-Unvalidated.

Eukaryotic cell lines

Policy information about cell lines and Sex and Gender in Research

Cell line source(s)	HEK293F (Thermo Fisher Scientific), Hela (ATCC), HEK293T (ATCC)
Authentication	STR profiling by ATCC
Mycoplasma contamination	Tested negative
Commonly misidentified lines<br>(See ICLAC register)	No commonly misidentified cell lines were used in the study
