# Supplementary material for: Ceramide sensing by human SPT-ORMDL complex for establishing sphingolipid homeostasis
Source: Nat Commun. 2023 Jun 13;14:3475. doi: 10.1038/s41467-023-39274-y (PMC10261145; doi:10.1038/s41467-023-39274-y)
Supplement: Supplementary file 4 — Source Data [file 41467_2023_39274_MOESM4_ESM.zip › Source_Data/Graphic_Source_data.pdf]

Fig.1b

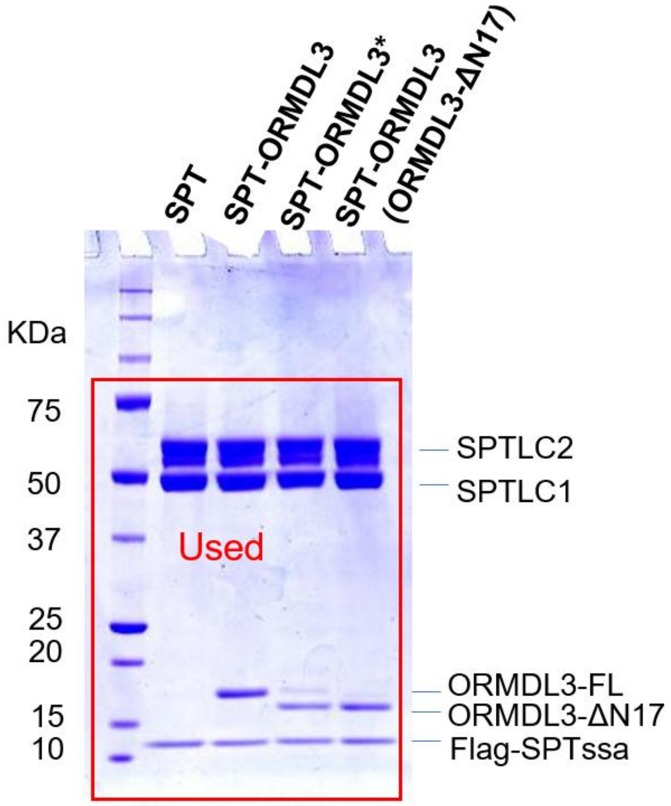
SPT
SPT-ORMDL3
SPT-ORMDL3*
SPT-ORMDL3
(ORMDL3-ΔN17)
KDa
75
50
37
25
20
15
10
Used
SPTLC2
SPTLC1
ORMDL3-FL
ORMDL3-ΔN17
Flag-SPTssa

Fig.6b

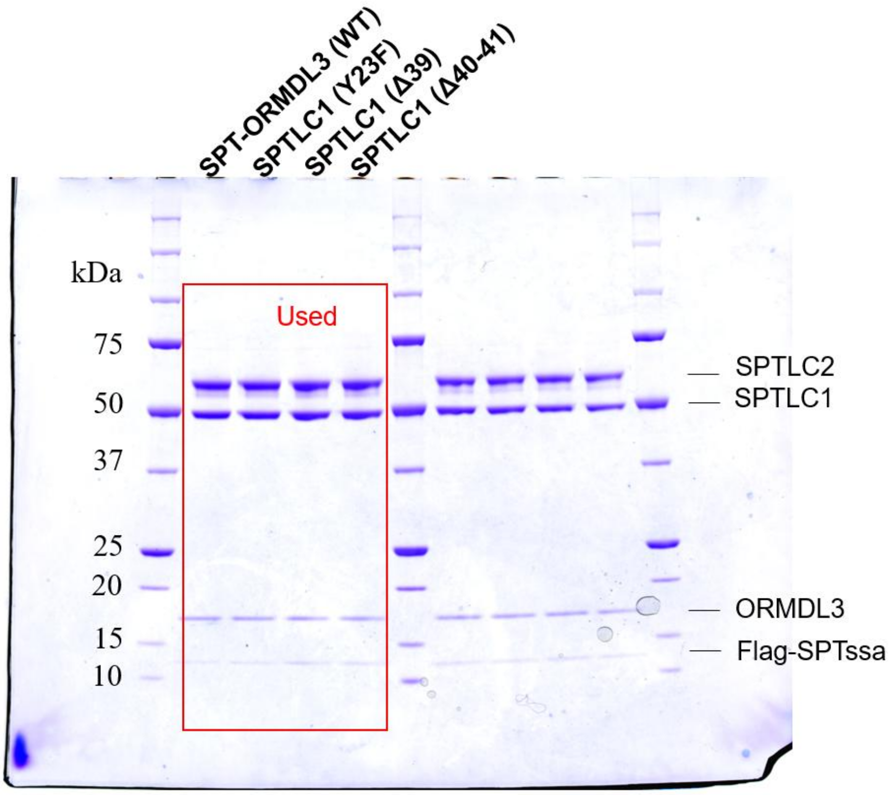
SPT-ORMDL3 (WT)
SPTLC1 (Y23F)
SPTLC1 (Δ39)
SPTLC1 (Δ40-41)
kDa
75
50
37
25
20
15
10
Used
SPTLC2
SPTLC1
ORMDL3
Flag-SPTssa

Supplementary Fig. 5e

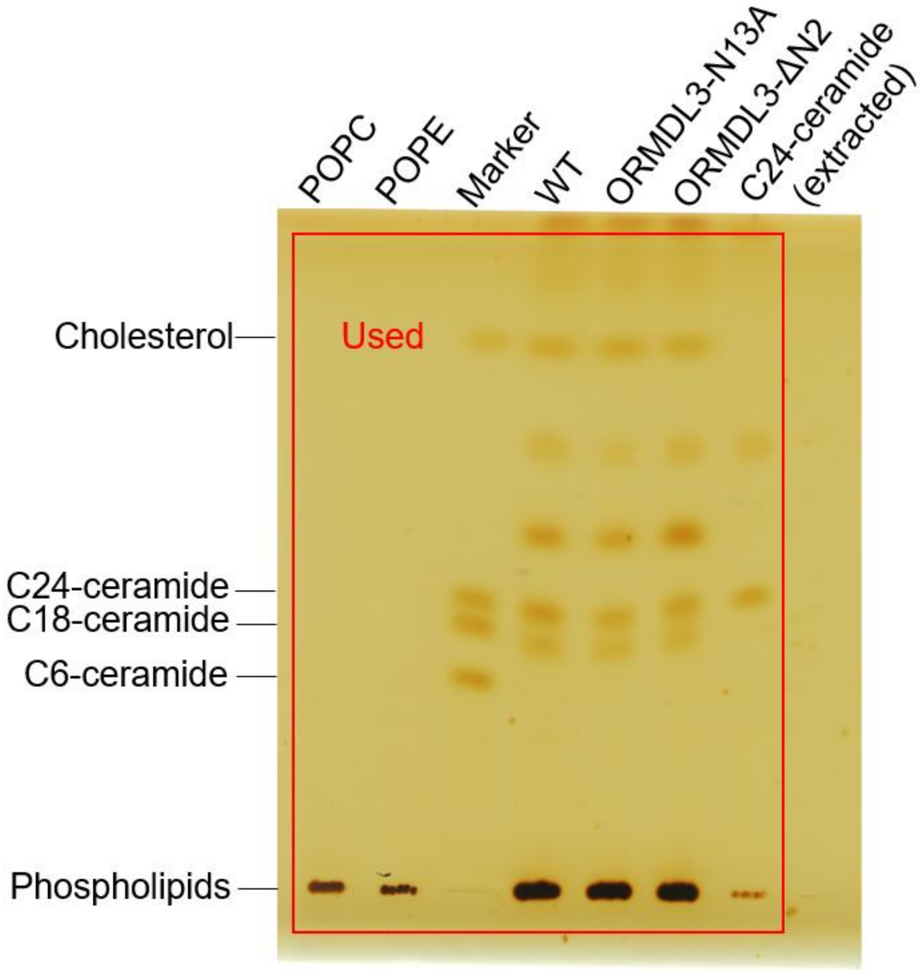

Supplementary Fig. 6a

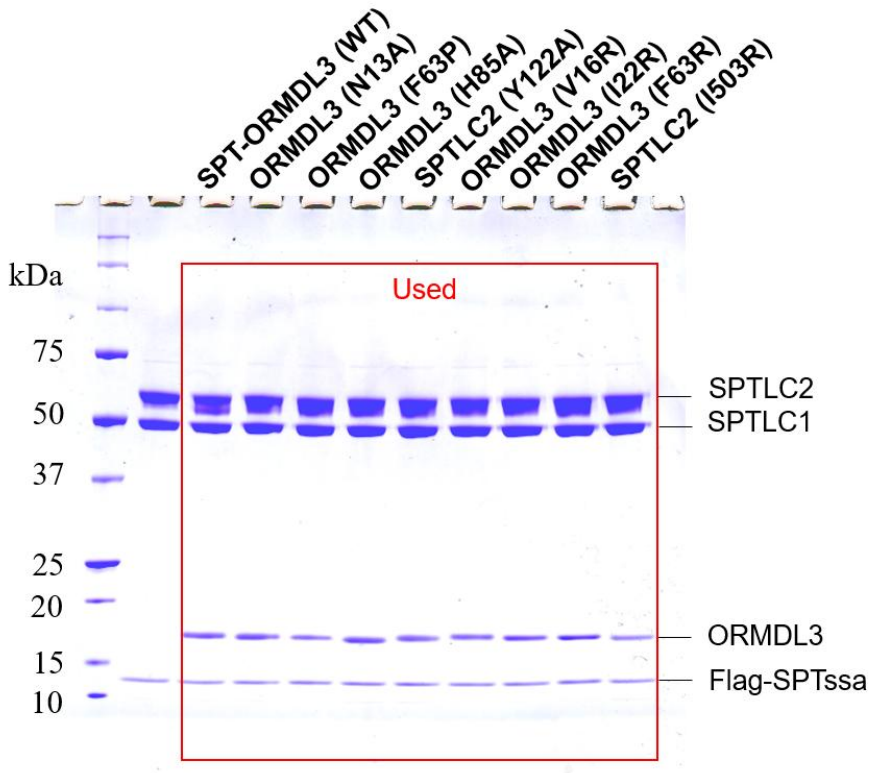

Supplementary Fig. 6b

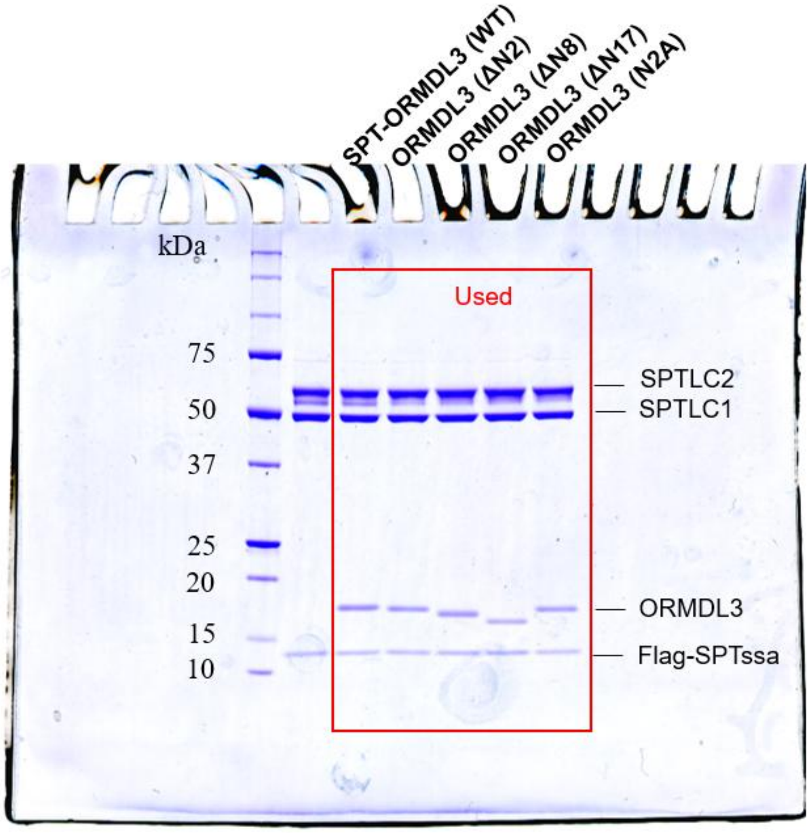

Supplementary Fig. 9a

Source File Supplementary Figure 9, Panel a. Left and right panels.

Right, bottom panels

Left, bottom panels

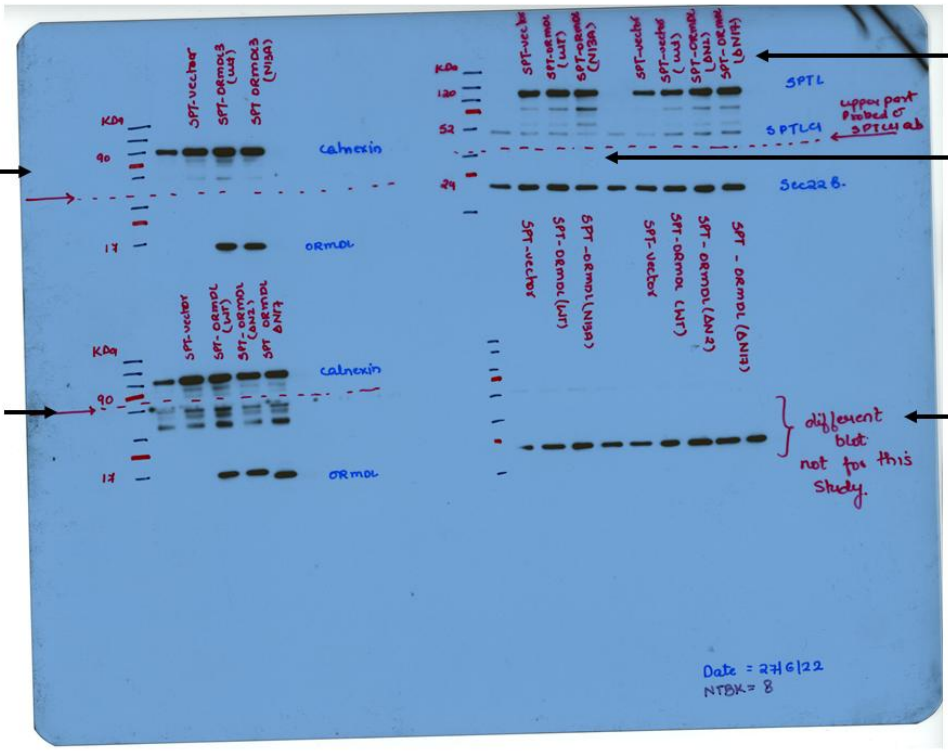

Left, top panels

Right, top panels

Not used in this study.

Source File Supplementary Figure 9, Panel a. Center panel.

Bottom panels

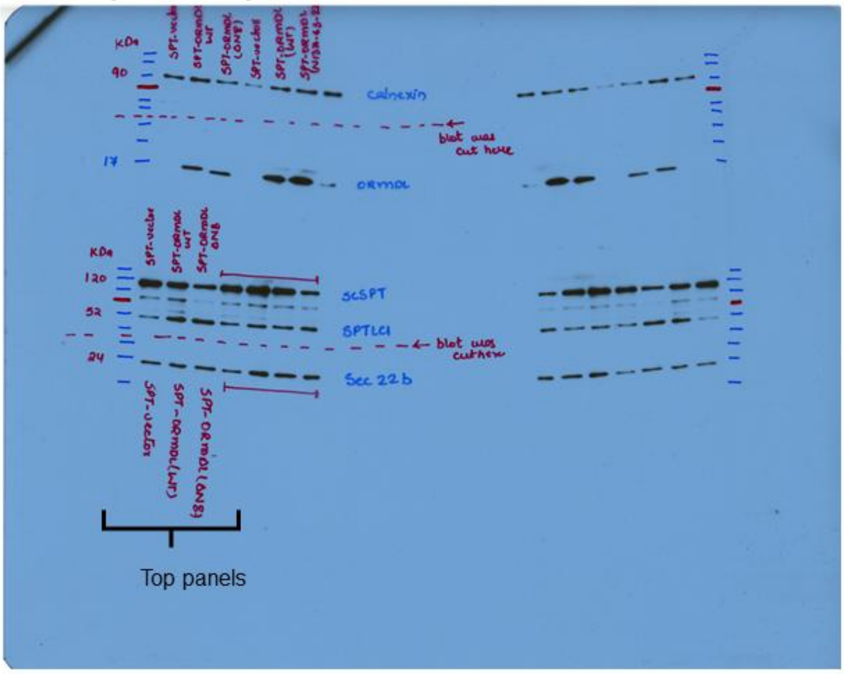

Top panels
